# Installation of authentic BicA and SbtA proteins to the chloroplast envelope membrane is achieved by the proteolytic cleavage of chimeric proteins in Arabidopsis

**DOI:** 10.1038/s41598-020-59190-1

**Published:** 2020-02-11

**Authors:** Susumu Uehara, Ayane Sei, Misaki Sada, Yasuko Ito-Inaba, Takehito Inaba

**Affiliations:** Department of Agricultural and Environmental Sciences, Faculty of Agriculture, University of Miyazaki, 1-1 Gakuenkibanadai-nishi, Miyazaki, 889-2192 Japan

**Keywords:** Biotechnology, Plant sciences

## Abstract

To improve the photosynthetic performance of C_3_ plants, installing cyanobacterial bicarbonate transporters to the chloroplast inner envelope membrane (IEM) has been proposed for years. In our previous study, we successfully introduced chimeric cyanobacterial sodium-dependent bicarbonate transporters, BicA or SbtA, to the chloroplast IEM of Arabidopsis. However, the installation of authentic BicA and SbtA to the chloroplast IEM has not been achieved yet. In this study, we examined whether or not tobacco etch virus (TEV) protease targeted within chloroplasts can cleave chimeric proteins and produce authentic bicarbonate transporters. To this end, we constructed a TEV protease that carried the transit peptide and expressed it with chimeric BicA or SbtA proteins containing a TEV cleavage site *in planta*. Chimeric proteins were cleaved only when the TEV protease was co-expressed. The authentic forms of hemagglutinin-tagged BicA and SbtA were detected in the chloroplast IEM. In addition, cleavage of chimeric proteins at the TEV recognition site seemed to occur after the targeting of chimeric proteins to the chloroplast IEM. We conclude that the cleavage of chimeric proteins within chloroplasts is an efficient way to install authentic bicarbonate transporters to the chloroplast IEM. Furthermore, a similar approach can be applied to other bacterial plasma membrane proteins.

## Introduction

Ribulose 1,5-bisphosphate carboxylase/oxygenase (Rubisco) catalyzes the incorporation of CO_2_ into ribulose 1,5-bisphosphate (RuBP), which is indispensable for carbohydrate production in plants. However, Rubisco also catalyzes the oxygenation reaction of RuBP. This reaction has been considered wasteful since extra energy is consumed to recover RuBP and CO_2_ is partially lost during the process of photorespiration. To compensate for the promiscuous nature of Rubisco, photosynthetic organisms have evolved various CO_2_-concentrating mechanisms (CCMs)^1,2^. For instance, cyanobacterial CCMs possess inorganic carbon (Ci) uptake systems and the microcompartments, carboxysomes, containing Rubisco. To date, five types of active Ci uptake systems have been identified in cyanobacteria. BicA and SbtA are single subunit sodium-dependent bicarbonate transporters on the plasma membrane^3,4^. In contrast, BCT1 is an ATP-binding, cassette-type bicarbonate transporter. The multimeric BCT1 complex is composed of four different subunits^5^. It has been proposed that the installation of CCM to chloroplasts is a promising approach to improve photosynthesis in C_3_ plants. According to a theoretical estimation, installing any one of the bicarbonate transporters, BicA, BCT1, or SbtA alone, to the chloroplast inner envelope membrane (IEM) may improve photosynthesis^6–8^.

It has been shown that chimeric cyanobacterial bicarbonate transporters expressed in the nucleus can be targeted to the chloroplast IEM^9,10^. Rolland *et al*. used a membrane protein leader (MPL) sequence that was fused to the N-terminus of the bicarbonate transporter together with the transit peptide^9^, which allowed the targeting of the MPL containing bicarbonate transporters to the chloroplast IEM in a transient assay. Likewise, we used the mature portion of an IEM protein, designated as Cor413im1, which contained an IEM targeting signal to deliver chimeric bicarbonate transporters to the chloroplast IEM^10–12^. This strategy achieved the expression of chimeric bicarbonate transporters in stable transgenic plants. However, those two studies also suggest that the precise targeting of cyanobacterial bicarbonate transporters to the chloroplast IEM requires IEM targeting signals that are uncleaved from the expressed protein. These observations are consistent with the fact that plastome-encoded authentic cyanobacterial bicarbonate transporters were not efficiently targeted to the chloroplast IEM^13^. Hence, it is challenging to install authentic forms of bicarbonate transporters to the chloroplast IEM.

One possible approach to install authentic bicarbonate transporters is to eliminate IEM targeting signals from the chimeric proteins *in vivo*. To remove a large tag portion during protein purification *in vitro*, tobacco etch virus (TEV) protease, which is a cysteine protease with stringent substrate specificity, has been used^14^. In an *in vitro* tandem affinity purification, the protein complex of interest can be cleaved by TEV protease from the tag protein that is immobilized on beads. In addition to *in vitro* applications, the protease has been shown to function in the mitochondria, peroxisomes, and cytosol of yeast cells^15,16^. Likewise, TEV protease expressed in mammalian secretory pathways was active^17^. It remains unclear if TEV protease targeted to chloroplasts can cleave substrate proteins *in vivo*. Nonetheless, in an *in vitro* cleavage assay, TEV protease was shown to cleave the chimeric BicA bicarbonate transporter on the isolated chloroplast IEM^10^. Taken together, the accumulating evidence suggests that the successful targeting of TEV protease together with chimeric bicarbonate transporters to chloroplasts is likely to eliminate portions other than authentic bicarbonate transporters.

In this study, we examined whether or not TEV protease targeted within chloroplasts can cleave chimeric proteins and produce authentic bicarbonate transporters. To this end, we took advantage of the cleavage of chimeric bicarbonate transporters by TEV protease *in vivo*. Using the transit peptide of the Rubisco small subunit, we successfully targeted TEV protease to the chloroplast stroma. The TEV protease within chloroplasts seemed to cleave the chimeric bicarbonate transporters on the chloroplast IEM, allowing the production of authentic bicarbonate transporters on the chloroplast IEM. Our results indicate that the *in vivo* cleavage of the chloroplast-targeted chimeric transporter proteins can serve as an effective way to introduce authentic transporter proteins derived from other organisms. This synthetic biology approach can therefore be used to accelerate the metabolic engineering of plants.

## Results

### Co-expression of chimeric bicarbonate transporters and TEV protease in Arabidopsis

**Figure 1:**
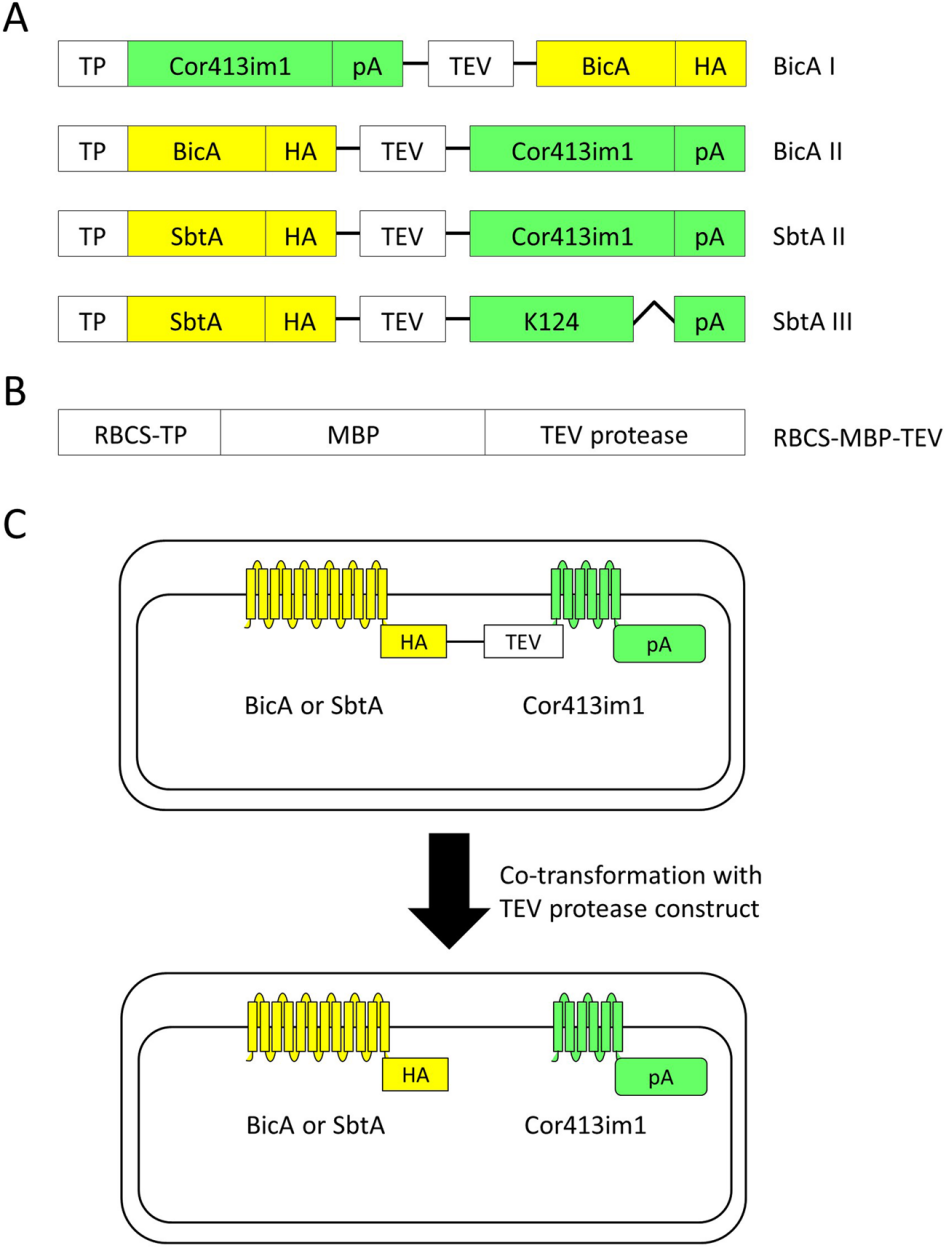
Construct designs for the chimeric bicarbonate transporters and tobacco etch virus (TEV) protease. (**A**) Schematic diagram of the chimeric BicA and SbtA constructs used in this study. The protein A domain (pA) of the fusion constructs contains two IgG-binding domains from staphylococcal protein A. The human influenza hemagglutinin (HA) domain consists of the amino acids YPYDVPDYA. Both BicA and SbtA genes are derived from *Synechocystis* sp. PCC 6803. The K124 construct lacks the 6th transmembrane domain of Cor413im1. TP, the transit peptide of Cor413im1; TEV, TEV recognition sequence (ENLYFQG). (**B**) Schematic diagram of TEV protease. The TEV protease gene is derived from the tobacco etch virus. RBCS–TP represents the transit peptide of the small subunit of Ribluse-1,5-bisphosphate carboxylase/oxygenase (Rubisco). MBP, Maltose binding protein. (**C**) Prediction before and after transformation with the TEV protease construct. When the bicarbonate transporter chimeric protein was co-expressed with TEV protease, we predicted that the TEV recognition sequence is digested by TEV protease in the chloroplast.

To install authentic bicarbonate transporters to the chloroplast IEM, we made a series of chimeric constructs possessing TEV protease cleavage site between the bicarbonate transporter portion and the other portion (Fig. 1). According to our previous study^12^, the topology of K124 has been shown to be flipped at the IEM, and the C-terminus faces toward the stroma. Hence, we assumed that the topology of a bicarbonate transporter fused to K124, SbtAIII, is reverted, as compared to that fused to the full-length Cor413im1, SbtAII^10^. To detect the chimeric proteins in the plants, hemagglutinin (HA) and protein A tags were added to each chimeric protein.

**Figure 2:**
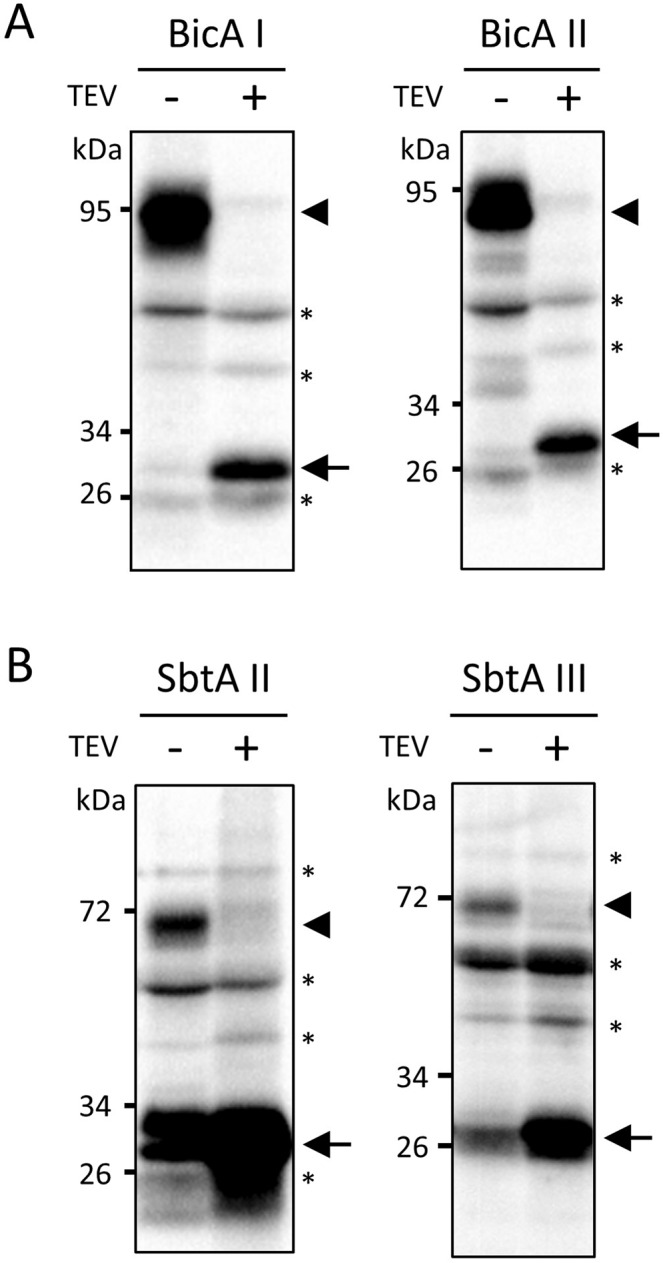
Expression analysis of chimeric BicA (**A**) and SbtA (**B**) with or without TEV protease in transgenic Arabidopsis. Total protein extracts (20 μg) from the rosette leaves were resolved by 12% SDS-PAGE and probed with antibodies against protein A. The arrowheads indicate the BicA– or SbtA–Cor413im1–protein A chimeric proteins. The arrows indicate the Cor413im1–protein A chimeric protein lacking a bicarbonate transporter. The asterisks indicate nonspecific proteins detected by the antibodies.

When each bicarbonate transporter alone was transformed into Arabidopsis, we observed the accumulation of each chimeric protein in the transformed Arabidopsis (Fig. 2, -TEV). The apparent molecular mass of each protein was quite similar to that observed for similar chimeric proteins in our previous study^10^. Next, we co-transformed each chimeric bicarbonate transporter construct and the TEV protease construct into the Arabidopsis plants. As shown in Fig. 1B, TEV protease was fused to the transit peptide of the Rubisco small subunit and maltose binding protein (MBP). The co-expression resulted in the appearance of a ~30 kDa fragment tagged with protein A (Fig. 2, +TEV). The apparent molecular masses of those proteins were close to the predicted molecular masses of the Cor413im1–protein A and K124–protein A portions. Probably due to poor reactivity of monoclonal antibody, we could not detect the HA-tagged BicA and SbtA bicarbonate transporters in the total extracts. Nonetheless, these data suggested that the chloroplast-targeted TEV protease was active within chloroplasts, thereby cleaving each chimeric protein into the transporter and Cor413im1 portions.

### Accumulation of authentic bicarbonate transporters on the chloroplast envelope membrane

**Figure 3:**
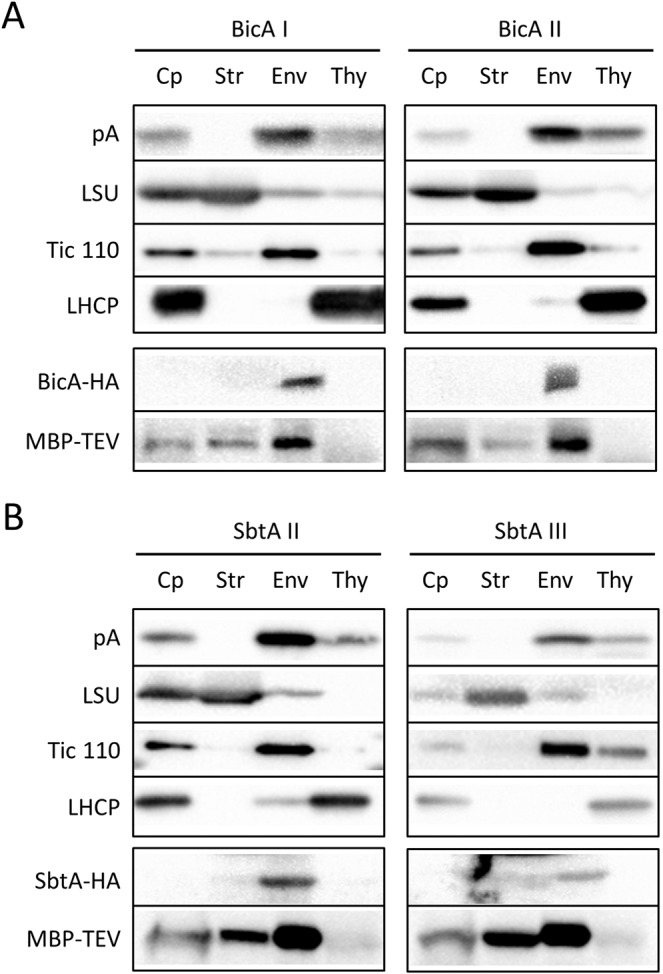
Localization of the chimeric proteins in the chloroplasts. Isolated chloroplasts (Cp) were fractionated into stroma (Str), envelope (Env), and thylakoid (Thy) fractions. The protein ratio of Cp to Str to Env to Thy used in these analyses was consistently 3: 3 :1:1.5. Each fraction was resolved by either 12% or 5–20% SDS-PAGE and immunoblotted with antibodies against protein A (pA; Cor413im1– and K124–protein A chimeric proteins), LSU, Tic110, LHCP, HA (HA-tagged authentic BicA and SbtA proteins), or MBP (MBP-fused TEV protease).

Next, we investigated whether the TEV-cleaved authentic bicarbonate transporters were targeted to the chloroplast envelope membrane. Intact chloroplasts were isolated from transgenic Arabidopsis plants expressing each transporter and the MBP-tagged TEV protease (MBP–TEV). Those chloroplasts were further fractionated into stroma, envelope, and thylakoid fractions. The purity of each fraction was confirmed using marker proteins such as the large subunit of Rubisco (LSU; stroma), Tic110 (envelope), and light-harvesting complex protein (LHCP; thylakoid). As shown in Fig. 3, all the cleaved Cor413im1–protein A and K124–protein A portions were localized within the chloroplasts. In addition, all those proteins were found to be enriched in the envelope membranes of the chloroplasts (Fig. 3A,B; lanes Env), indicating that they were localized to the envelope membranes of the chloroplasts.

We next investigated if the authentic bicarbonate transporters also accumulated in the chloroplast envelope membrane. As summarized in Fig. 1C, each bicarbonate transporter is supposed to be tagged with HA but not with protein A after cleavage by TEV protease. Using this principle, we detected authentic bicarbonate transporters using an anti-HA monoclonal antibody. The HA-tagged BicA and SbtA bicarbonate transporters were undetectable in the total chloroplast fractions, probably due to their lower abundance or poor reactivity of antibody (Fig. 3). However, both HA-tagged BicA and SbtA transporters were highly enriched in the envelope fractions and became detectable (Fig. 3A,B; BicA–HA and SbtA–HA).

We also examined the localization of TEV protease within chloroplasts. The MBP–TEV was fused to the transit peptide of Rubisco (Fig. 1B). Hence, we anticipated that the MBP–TEV localized to the chloroplast stroma. The MBP–TEV localized in the chloroplast stroma but also associated with the envelope membrane (Fig. 3). The distribution pattern of the MBP–TEV was quite similar to that of acetyl-CoA carboxylase, a stromal enzyme known to associate with the inner envelope membrane^18–20^.

These data indicated that the transgenic plants co-expressing the bicarbonate transporters and TEV protease successfully accumulated authentic bicarbonate transporters on the chloroplast envelope membranes.

### Authentic bicarbonate transporters are produced by the cleavage of chimeric proteins on the chloroplast inner envelope membrane

**Figure 4:**
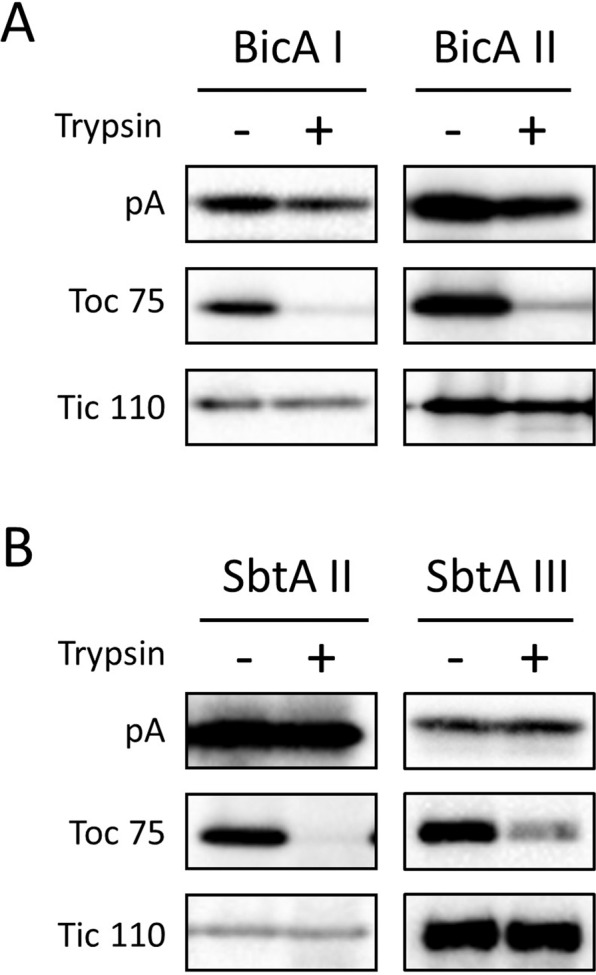
Trypsin sensitivity of BicA (**A**) and SbtA (**B**) chimeric proteins in the intact chloroplasts. Chloroplasts, equivalent to 25 μg of chlorophyll, were treated with trypsin on ice for 30 min. The trypsin was inactivated and the intact chloroplasts were re-isolated, resolved by either 12% or 5–20% SDS-PAGE, and immunoblotted with the antibodies against protein A (pA). The protease sensitivities of the outer envelope membrane protein, Toc75, and the inner envelope membrane protein, Tic110, were included as a positive and negative control to confirm the validity of the experiments, respectively.

We next investigated whether each authentic protein is an outer envelope membrane (OEM) or IEM protein. We isolated intact chloroplasts from each transgenic plant and treated them with trypsin. Trypsin permeates the OEM, but not the IEM, of intact chloroplasts. The validity of the trypsin treatment was confirmed by the fact that Toc75, an OEM protein, was digested by trypsin while Tic110, an IEM protein, was resistant to trypsin treatment (Fig. 4, Tic110). However, as expected and shown in Fig. 3, we were unable to detect the HA-tagged bicarbonate transporters in the trypsin-treated and -untreated chloroplast fractions. Instead, we investigated whether each Cor413im1-containing portion was an OEM or IEM protein. Both the cleaved Cor413im1–protein A and K124–protein A portions have been shown to localize on the chloroplast envelope membranes (Fig. 3). Hence, trypsin treatment can directly address the question of whether those proteins are located on the chloroplast IEM. As shown in Fig. 4, all the protein A-tagged portions derived from the chimeric proteins were protected from trypsin, indicating that they were located on the chloroplast IEM.

**Figure 5:**
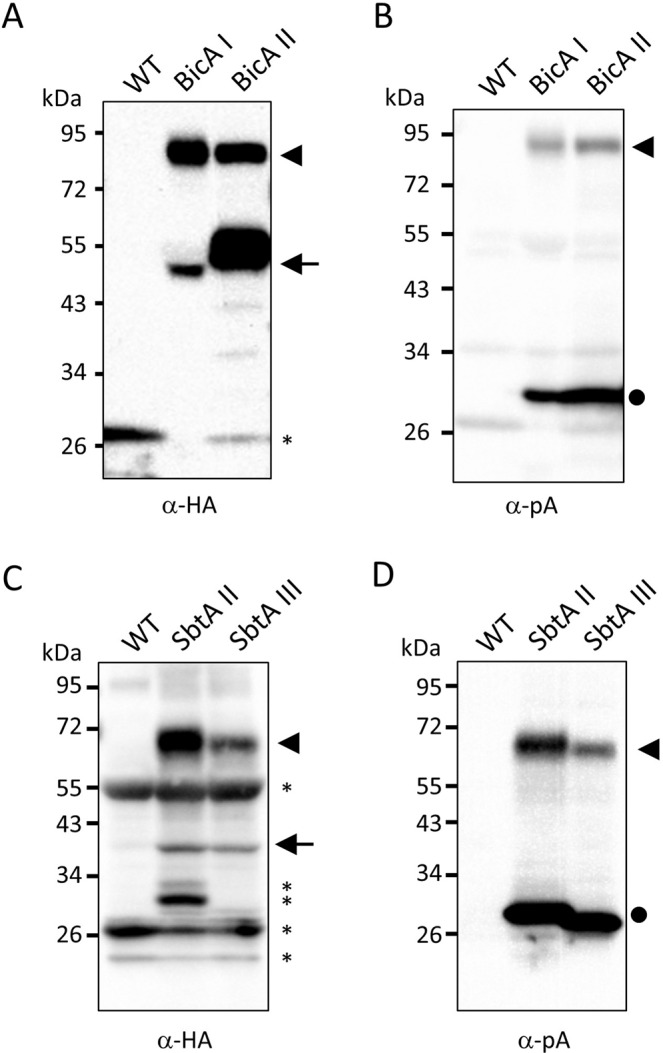
Detection and comparison of HA- and protein A-tagged proteins in the chloroplast envelope membranes. Envelope fractions (14 μg for panels **A** and **B**, and 20 μg for panels **C** and **D**) were resolved by 5–20% SDS-PAGE and immunoblotted with antibodies against HA (**A,C**) or protein A (pA; **B,D**). The arrowheads indicate the full-length BicA– or SbtA–Cor413im1–protein A chimeric proteins. The arrows indicate the HA-tagged BicA and SbtA proteins. The closed circles indicate the Cor413im1–protein A and K124–protein A chimeric proteins. The asterisks indicate nonspecific proteins and degradation products detected by the antibodies.

In our previous study, we demonstrated that the BicAI, BicAII, SbtAII, and SbtAIII chimeric proteins were localized on the IEM (Uehara *et al*.^10^). The fact that the Cor413im1–protein A and K124–protein A portions derived from those chimeric proteins were also located on the IEM (Fig. 4) strongly suggests the localization of authentic bicarbonate transporters on the IEM. To further prove this hypothesis, we investigated the co-distribution of the full-length chimeric protein, the Cor413im1–protein A (or K124–protein A) portion, and the authentic bicarbonate transporter using isolated envelope membranes. When the envelope membranes isolated from BicAI and BicAII were resolved by SDS-PAGE and probed with an antibody against the HA tag, two bands were detected in each sample (Fig. 5A). One band was the full-length chimeric protein (approximately 90 kDa), and the other band was the authentic bicarbonate transporter protein (approximately 60 kDa). The full-length chimeric proteins were also detected by antibodies against protein A (Fig. 5B), but the authentic bicarbonate transporters were undetectable. Instead, a protein of approximately 30 kDa, which corresponded to the Cor413im1–protein A, was detected by anti-protein A antibodies (Fig. 5B). Similar results were obtained from the SbtAII and SbtAIII plants (Fig. 5C,D). Taken together, these data suggest that the chimeric proteins were cleaved by TEV protease on the chloroplast IEM and that the authentic bicarbonate transporters are located on the chloroplast IEM.

## Discussion

It has been proposed that the introduction of cyanobacterial bicarbonate transporters into the chloroplasts of land plants is a powerful tool to improve photosynthesis and crop yield^6–8,21^. In previous studies, we and others have successfully targeted cyanobacterial sodium-dependent bicarbonate transporters, BicA and SbtA, to the chloroplast IEMs of Arabidopsis and tobacco^9,10^. However, such studies have used chimeric bicarbonate transporters that were fused to a large tag, such as GFP and protein A. Therefore, the installation of authentic cyanobacterial bicarbonate transporters to the chloroplast IEM of stably transformed land plants have not been achieved to date. In this study, we examined whether or not TEV protease targeted within chloroplasts can cleave chimeric proteins and produce authentic bicarbonate transporters. TEV protease that was fused to the transit peptide of Rubisco was successfully targeted to the interior of chloroplasts, and it cleaved the chimeric proteins (Fig. 2). Furthermore, the authentic bicarbonate transporters cleaved from the chimeric protein resided in the chloroplast IEM (Figs. 3–5). Overall, we established a novel method that allows the installation of authentic bicarbonate transporters to the chloroplast IEM.

Because of its high specificity, TEV protease has been used to purify TAP-tagged protein complexes from various organisms^14^. However, as far as we know, whether TEV protease can function within chloroplasts remains unclear. We showed that chloroplast-targeted TEV protease can cleave chimeric proteins, allowing the production of authentic bicarbonate transporters *in vivo* (Fig. 2). In a previous study, the expression of authentic bicarbonate transporters from the chloroplast genome failed to target transporters to the chloroplast IEM efficiently^13^. While the chloroplast encoded protein was targeted from the stroma to the IEM via a reinsertion mechanism, the majority of nuclear encoded proteins utilized the stop transfer mechanism for their targeting to the chloroplast IEM^22,23^. Hence, a major challenge has been to target membrane proteins expressed from the chloroplast genome to the chloroplast IEM. Our results indicate that the co-expression of the chloroplast-targeted TEV protease and the chimeric proteins containing TEV cleavage sites in the nucleus is likely to overcome the difficulty of installing bacterial membrane proteins to the chloroplast IEM.

The mechanism by which chimeric proteins are cleaved by TEV protease remains to be characterized. To date, two distinct pathways have been shown to be involved in targeting to the IEM proteins. One is the stop transfer pathway, and the other is the post-import or conservative pathway. According to a previous study, Cor413im1 seems to utilize the stop transfer pathway for its targeting to the chloroplast IEM^12^. Given the fact that the full-length chimeric protein, the HA-tagged bicarbonate transporter, and the Cor413im1–protein A portion were all found in IEM fraction, it is conceivable to speculate that the cleavage of the chimeric bicarbonate transporters occurs on the IEM after their targeting. Therefore, TEV protease in the stroma is capable of splitting chimeric membrane proteins into two portions. This property of TEV protease can be further utilized in other organelles. We can transform chimeric genes of interest carrying TEV recognition sites and organelle targeting signals together with TEV protease. Organelle-targeted TEV protease cleaves chimeric proteins to produce authentic proteins of our interest, making it possible to produce various membrane proteins in various organelles in their authentic form. As such, our findings would have significant impacts on metabolic engineering accompanied by membrane protein expression in plants.

**Figure 6:**
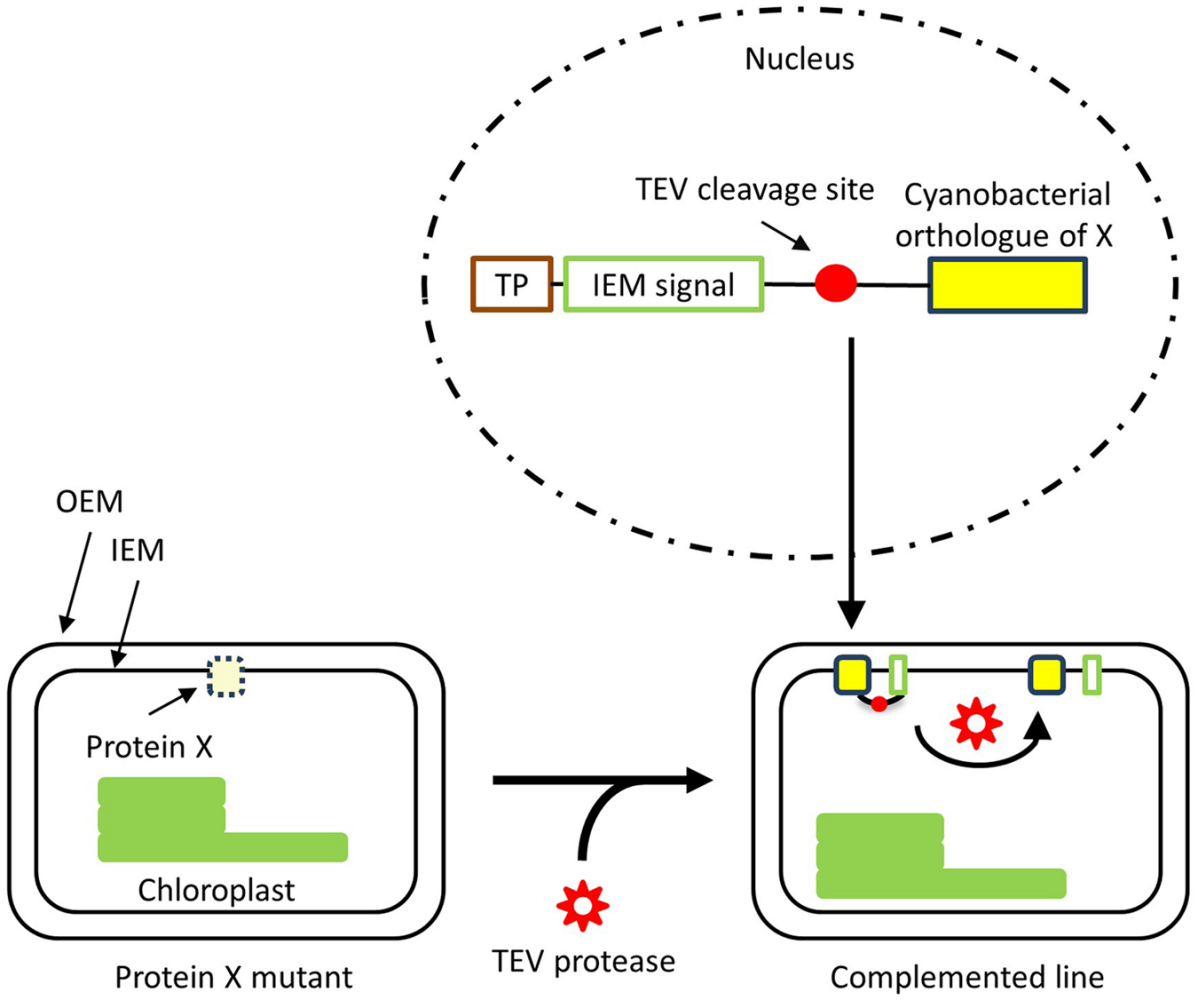
Proposed model for the complementation test of a mutant plant lacking an IEM protein using a cyanobacterial orthologue. A nuclear-encoded cyanobacterial orthologue carrying a transit peptide (TP) and IEM targeting signal (IEM signal) is expected to be targeted to the IEM of chloroplasts. Then, TEV protease should cleave the chimeric protein, allowing the accumulation of the cyanobacterial orthologue on the chloroplast IME.

Our approach can be further applied to investigate the origin of chloroplast envelope proteins. Since chloroplasts originated from a cyanobacterial ancestor, there are a number of cyanobacterial plasma membrane proteins that are likely to be orthologous to chloroplast IEM proteins^24,25^. However, a systematic approach to analyze those cyanobacterial orthologues using land plants have not been established yet. Our results established the method to introduce authentic cyanobacterial plasma membrane proteins into the chloroplast IEM. If the DNA construct is optimized, any protein of the cyanobacterial plasma membrane is likely to be targeted to the chloroplast IEM using the same method. Hence, our method may be applied to perform functional complementation in mutants lacking IEM proteins using cyanobacterial orthologues, which may help us to further understand the roles of cyanobacterial plasma membrane proteins and chloroplast IEM proteins (Fig. 6).

In summary, we successfully installed authentic cyanobacterial bicarbonate transporters, BicA and SbtA, to the chloroplast IEM. The fact that authentic cyanobacterial bicarbonate transporters could be targeted to the chloroplast IEM will further accelerate the full reconstitution of bacterial CO_2_-concentrating mechanisms in chloroplasts.

## Methods

### Construction of the vector and Arabidopsis transformation

The construction of the BicA and SbtA genes are summarized in Supplementary Fig. S1. All the fragments used for DNA construction were amplified by PCR using KOD DNA polymerase (TOYOBO). The primers used to amplify each portion are listed in Supplementary Table S1. Multiple fragments were subcloned into the *Nco*I–*Nhe*I sites of pCAMBIA1301 using an In-Fusion HD Cloning Kit (Takara) to create each BicA or SbtA construct.

For the construction of RBCS–MBP–TEV, the gene encoding TEV protease fused to MBP was amplified by PCR using the pRK1043 plasmid as the template. Meanwhile, the coding sequence for the transit peptide plus the first eight residues of the mature portion of RBCS1B were amplified by PCR. Those two fragments were subcloned simultaneously into the pUC19 vector containing a CaMV35S promoter–NOS terminator cassette. This allowed the creation of the MBP–TEV gene fused to the transit peptide of RBCS1B. The resulting construct, CaMV35S promoter–RBCS–MBP–TEV–NOS terminator, was further amplified by PCR and sub-cloned into the XbaI site of pCAMBIA1301 containing the BicA or SbtA construct using an In-Fusion HD Cloning Kit (Takara) to obtain the constructs summarized in Fig. 1A.

All pCAMBIA constructs were introduced into *Arabidopsis thaliana* (accession Columbia) via *Agrobacterium tumefaciens*-mediated transformation using the floral dip method^26^.

### Plant material and growth conditions

Wild type (WT, accession Columbia) and transgenic plants expressing chimeric BicA or SbtA proteins were grown at 22 °C under long day or continuous light conditions (16 h light to 8 h dark or 24 h light; light intensity, 100–120 μphotons m^−2^ s^−1^).

### Arabidopsis chloroplast isolation and membrane fractionation

For chloroplast isolation, Arabidopsis plants were grown on 0.5× MS plates supplemented with 1% sucrose. Chloroplasts were isolated from 14- to 18-day-old transgenic plants as described previously^27,28^.

### Analysis of the localization of each chimeric protein within the chloroplasts

To determine the localization of each chimeric protein within the chloroplasts, isolated chloroplasts were fractionated into stroma, envelope, and thylakoid membranes as described previously^27,28^. After the quantification of proteins in each fraction, the total chloroplast (3 μg), stroma (3 μg), envelope (1 μg), and thylakoid (1.5 μg) fractions were analyzed by sodium dodecyl sulfate polyacrylamide gel electrophoresis (SDS-PAGE) using 12% or 5–20% polyacrylamide gel, and immunoblotted with the antisera indicated in the figures. Although we sometimes loaded a different amount of protein in the analysis, the protein ratio of total chloroplast to stroma to envelope to thylakoid was consistently 3:3:1:1.5. The trypsin sensitivity of the chimeric BicA and SbtA proteins was examined using intact chloroplasts as described previously^28–30^.

The antibodies against LSU, Tic110, and Toc75 have been previously described^10,12,28,31–33^. The LHCP antibodies were a kind gift from Prof. Kenneth Cline. The anti-protein A IgG was purchased from Sigma-Aldrich. The anti-HA monoclonal antibody was purchased from Roche. The anti-MBP monoclonal antibody was purchased from Medical and Biological Laboratories (Nagoya, Japan). Signals were detected using horseradish peroxidase-conjugated secondary antibodies and chemiluminescence reagent. All the uncropped blots were shown in Supplementary Figs. S2 and S3.

### Measurement of protein and chlorophyll concentrations

Chlorophyll and protein concentrations were quantified as described elsewhere^28^.

## Supplementary information

Supplementary Information.
