# Supplementary material for: Installation of authentic BicA and SbtA proteins to the chloroplast envelope membrane is achieved by the proteolytic cleavage of chimeric proteins in Arabidopsis
Source: Sci Rep. 2020 Feb 11;10:2353. doi: 10.1038/s41598-020-59190-1 (PMC7012931; doi:10.1038/s41598-020-59190-1)
Supplement: Supplementary file 1 — Supplementary Information. [file 41598_2020_59190_MOESM1_ESM.pdf]

Supplementary Information

Installation of authentic BicA and SbtA proteins to the chloroplast envelope membrane is achieved by the proteolytic cleavage of chimeric proteins in Arabidopsis

Authors
Susumu Uehara[1,2], Ayane Sei[1,2], Misaki Sada[1], Yasuko Ito-Inaba[1] and Takehito Inaba[1,*]

[1]Department of Agricultural and Environmental Sciences, Faculty of Agriculture, University of Miyazaki, 1-1 Gakuenkibanadai-nishi, Miyazaki 889-2192, Japan
[2]These authors contributed equally to this work

[*]Corresponding author
Takehito Inaba
Tel/Fax: +81-985-58-7899
E-mail: tinaba@cc.miyazaki-u.ac.jp

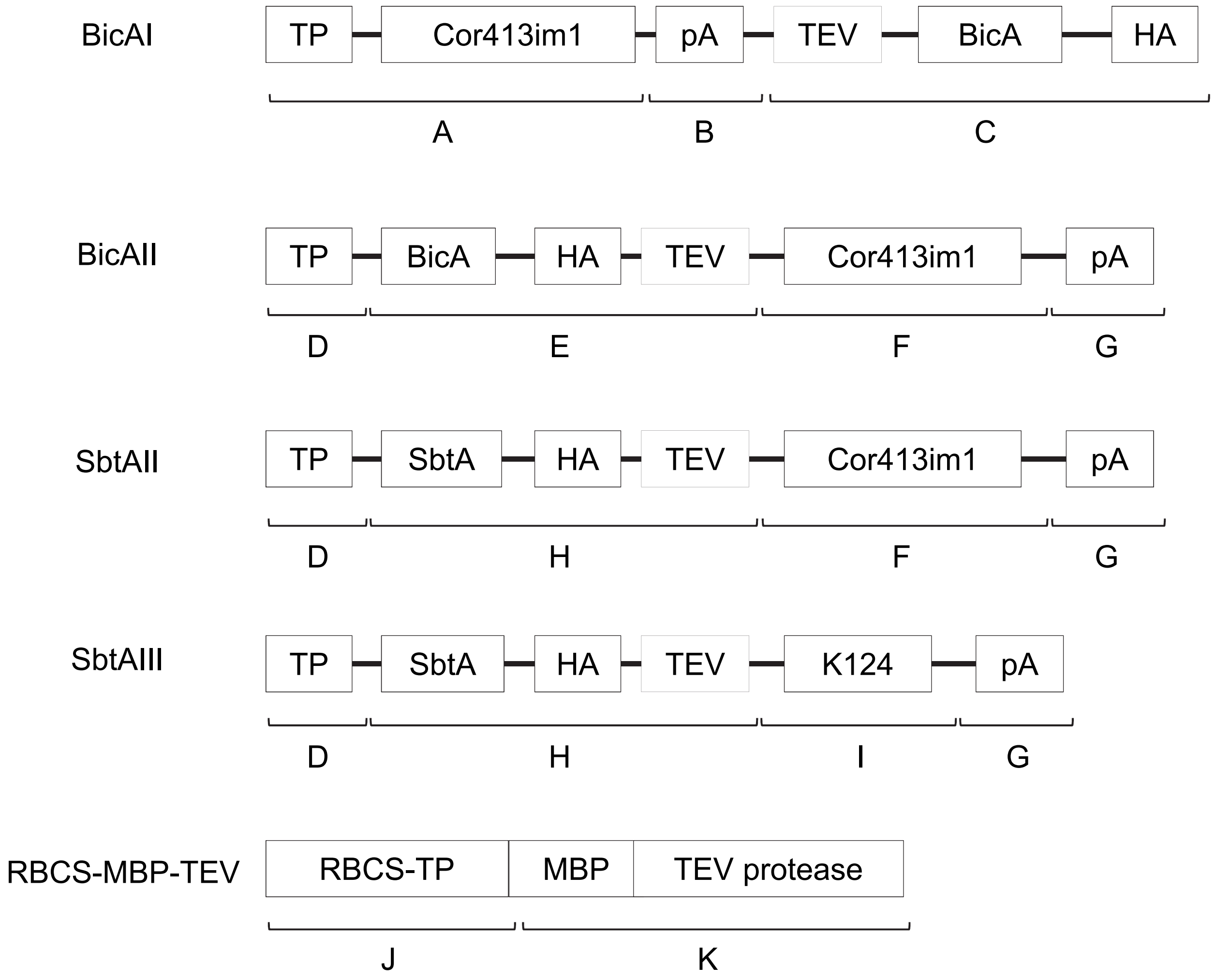

Supplementary Figure S1. Overview of the constructs used in this study.
Fragments A to K were separately amplified by PCR using the primers listed in Supplementary Table 1. The amplified fragments were subcloned into vectors as described in the methods.
pA, two IgG-binding domains from staphylococcal protein A; HA, human influenza hemagglutinin tag (YPYDVPDYA); BicA and SbtA, bicarbonate transporters derived from *Synechocystis* sp. PCC 6803; K124, the construct lacking the 6th transmembrane domain of Cor413im1; TP, the transit peptide of Cor413im1; TEV, TEV protease recognition sequence (ENLYFQG); RBCS-TP, the transit peptide of the Rubisco small subunit.

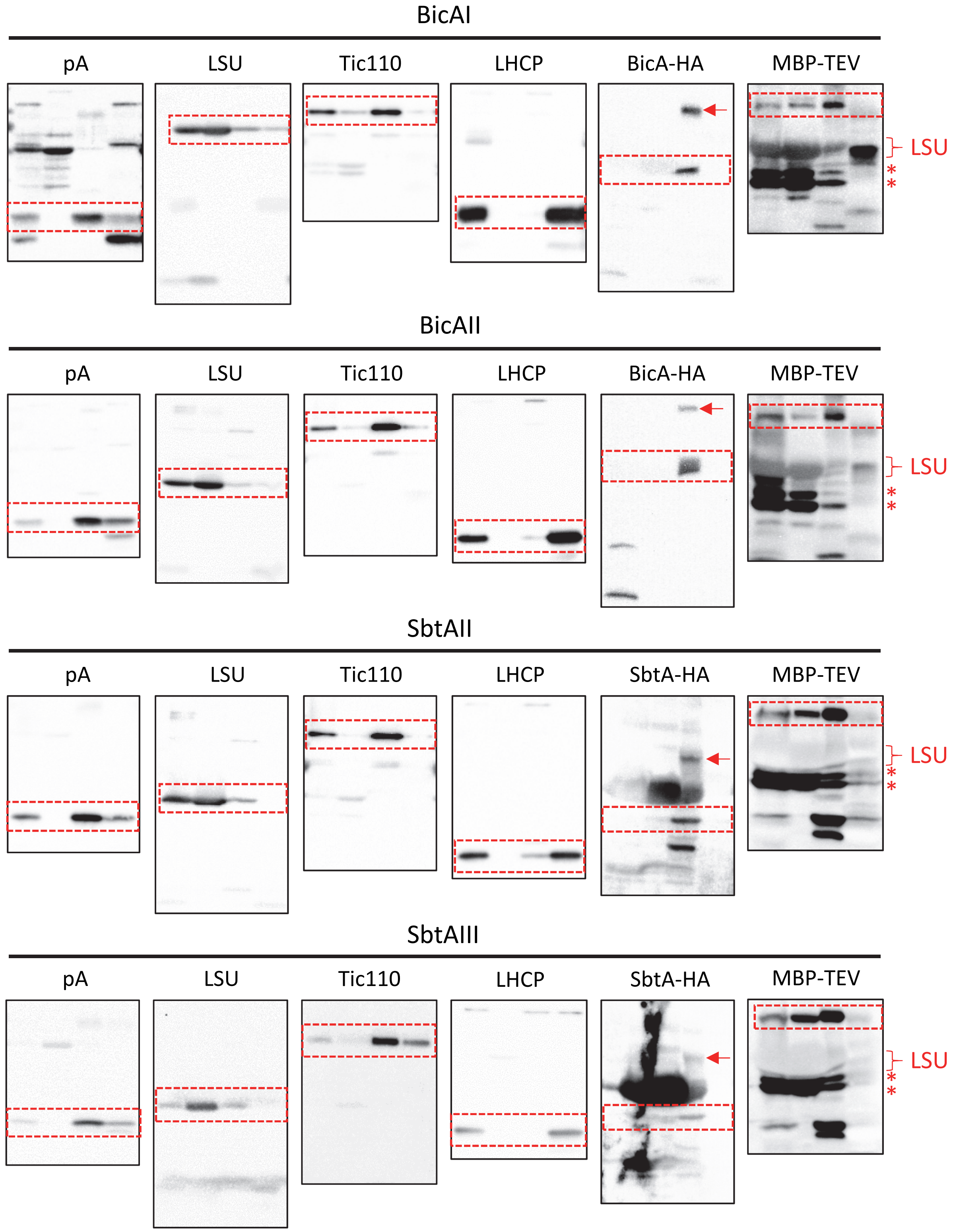

Supplementary Figure S2. Uncropped western blot images for Figure 3.
Dashed boxes indicate areas that were cropped. Arrows indicate uncleaved version of chimeric proteins. Asterisks indicate degradation products of MBP-TEV detected by anti-MBP monoclonal antibody. LSU indicates the location of large subunit of Rubisco on the MBP-TEV membrane.

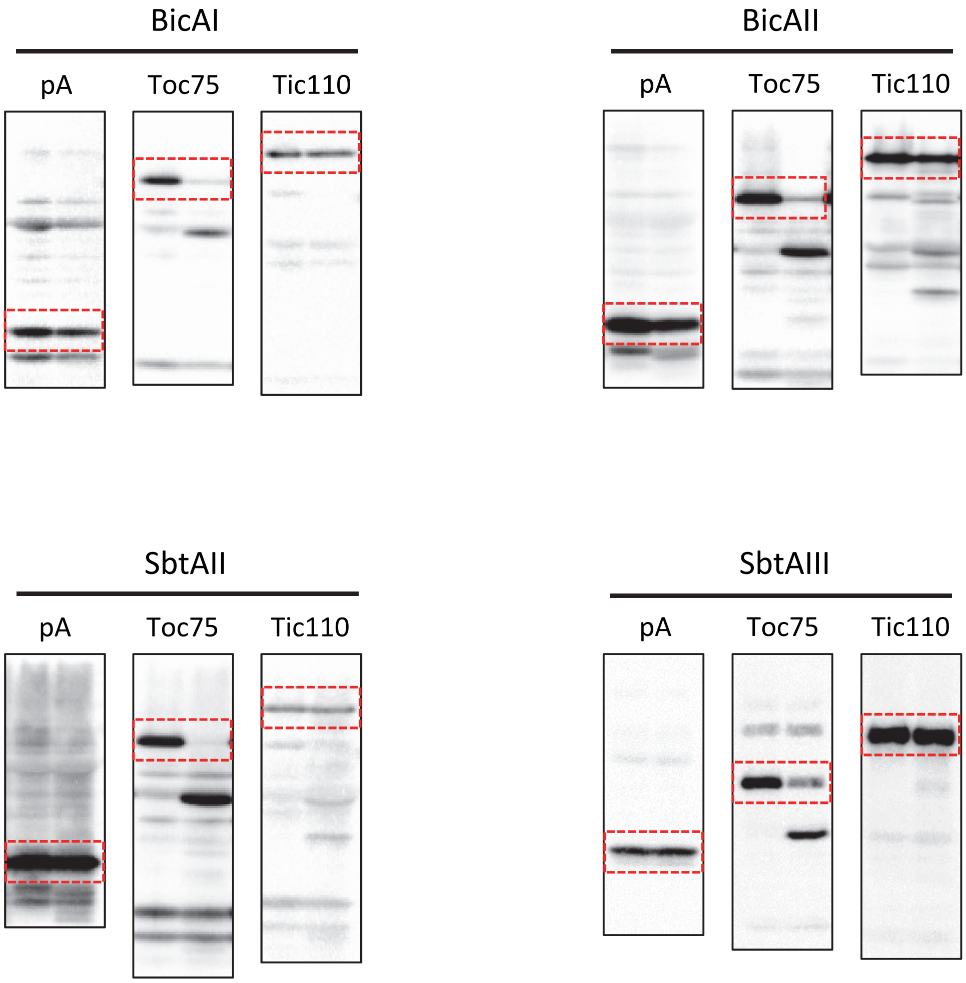

Supplementary Figure S3. Uncropped western blot images for Figure 4.
Dashed boxes indicate areas that were cropped.

Supplementary Table S1. Primers used to construct the chimeric bicarbonate transporters and TEV protease

Fragment	Forward	Reverse
A	GGACTCTTGACCATGGCGAGTCTCTGTCTCT CATCG	GTCGAGAGGGATGTCGTAGTCTGATCCACCA CTGCCTCCTAGCAACAAAAGGAGCGATAC
B	GACATCCCTCTCGACCCGAAAGCTGATAACA AT	TCCCTGGAAGTACAGGTTCTCTTTTGGTGCTT GAGCAT
C (1st round)	CTGTACTTCCAGGGAATGCAAATAACTAACAA AATTCAT	GTAATCTGGAACATCGTATGGGTAGTATGTG GTCTGGACGGAAGA
C (2nd round)*	CTGTACTTCCAGGGAATGCAAATAACTAACAA AATTCAT	GGTGGTGGTGGCTAGCTCAAGCGTAATCTGG AACATCGTATGG
D	GGACTCTTGACCATGGCGAGTCTCTGTCTCT CATCG	GCGGCCGCAAGCTTGTCGACG
E (1st round)	CAAGCTTGCGGCCGCATGCAAATAACTAACA AAATTCAT	GTAATCTGGAACATCGTATGGGTAGTATGTG GTCTGGACGGAAGA
E (2nd round)*	CAAGCTTGCGGCCGCATGCAAATAACTAACA AAATTCAT	TCCACCACTGCCTCCTCCCTGGAAGTACAGG TTCTCAGCGTAATCTGGAACATCGTATGG
F	GGAGGCAGTGGTGGATCAGCTGCT	GTCGAGAGGGATGTCGTAGTCTGATCCACCA CTGCCTCCTAGCAACAAAAGGAGCGATAC
G	GACATCCCTCTCGACCCGAAAGCTGATAACA AT	GGTGGTGGTGGCTAGCTCATTTTGGTGCTTG AGCAT
H (1st round)	CAAGCTTGCGGCCGCATGGATTTTTTGTCCA ATTTCTTG	GTAATCTGGAACATCGTATGGGTAACCTGCA CCAAGGGTCTGGGC
H (2nd round)*	CAAGCTTGCGGCCGCATGGATTTTTTGTCCA ATTTCTTG	TCCACCACTGCCTCCTCCCTGGAAGTACAGG TTCTCAGCGTAATCTGGAACATCGTATGG
I	GGAGGCAGTGGTGGATCAGCTGCTCCGATTT CTGCCAAT	GTCGAGAGGGATGTCGTAGTCTGATCCACCA CTGCCTCCTTTCTCCAAGCTCCCTGCTCG
J	CAGGGGGGACTCTAGATGGCTTCCTCTATGC TCTCCTCT	TTCTTCGATTTTCATTTCGACGTCAGTAAGGT CAGGGAG
K	CTTACTGACGTCGAAATGAAAATCGAAGAAG GTAAACTG	GATCGAATTCTCTAGTTAGCGACGGCGACGA CGATTCAT

* Fragments C, E, and H were amplified by a two-step PCR process. After a portion of each fragment was amplified by the first round of PCR, the entire fragment was amplified by performing the second PCR using the first round PCR product as the template.
